# Radiotherapy for Metastatic Bladder Cancer in the Era of Modern Systemic Therapies

**DOI:** 10.7759/cureus.113796

**Published:** 2026-08-01

**Authors:** Martin E Herver Vazquez, Maria J Mora Montoya, Luis A Coria Dominguez, Erick F Granados Sanchez

**Affiliations:** 1 Radiation Oncology, Centro Estatal de Cancerología "Dr. Miguel Dorantes Mesa", Xalapa, MEX; 2 Radiation Oncology, Universidad Veracruzana, Xalapa, MEX; 3 Medical Physics, Centro Estatal de Cancerología "Dr. Miguel Dorantes Mesa", Xalapa, MEX

**Keywords:** bladder adenocarcinoma, case review, consolidative radiotherapy, immunotherapy, oligo-metastasis, oligometastatic disease

## Abstract

The management of metastatic bladder cancer is transitioning from a purely palliative model toward multimodal strategies, especially in the setting of oligometastatic disease. While systemic therapies, such as immunotherapy, have improved survival, the role of consolidative radiotherapy (RT) remains a subject of ongoing research, particularly in rare entities such as primary bladder adenocarcinoma. This review analyzes the integration of high-precision RT with modern systemic treatments to achieve durable disease control. We present the case of a male patient with stage IV bladder adenocarcinoma and synchronous brain and pulmonary metastases. Initial management involved whole-brain RT (30 Gy in 10 fractions) because of the intracranial tumor burden, followed by systemic immunotherapy with pembrolizumab. After achieving systemic stability and transitioning to an oligopersistent state, the patient received radical consolidative RT to the primary bladder tumor (50.4 Gy in 28 fractions). Subsequent consolidative surgical resection was performed, with histopathology confirming a complete pathologic response (ypT0N0). At the latest follow-up, the patient remains asymptomatic, with no evidence of active systemic disease while continuing maintenance immunotherapy. This case illustrates that a sequential, radical approach integrating RT, immunotherapy, and surgery is feasible and can lead to prolonged survival in selected patients with metastatic bladder adenocarcinoma. The shift toward consolidative local therapies in the era of modern systemic treatments represents a significant paradigm change, suggesting that RT is no longer limited to palliation but is a vital tool for achieving long-term oncologic control.

## Introduction

The management of metastatic cancer has evolved substantially, transitioning from a predominantly palliative model toward personalized and sequential therapeutic strategies in patients with oligometastatic disease, defined as the presence of a limited number of metastatic lesions potentially amenable to local treatment with radical intent, either alone or in combination with other therapies [1]. Traditionally, radiotherapy (RT) was used with palliative intent to relieve symptoms resulting from metastatic disease or to achieve local control of metastatic lesions [2]. However, the development of more effective systemic therapies, such as immunotherapy, together with advances in high-precision RT techniques, has modified this approach. Currently, in selected patients with adequate systemic control and prolonged survival, consolidative strategies and, in highly selected cases, approaches aimed at prolonged disease control may be considered.

Nevertheless, the application of this strategy presents a particular challenge in primary bladder adenocarcinoma, an uncommon entity. The scarcity of high-level evidence, mainly limited to retrospective series, together with the need to extrapolate recommendations from guidelines developed for urothelial carcinoma, generates considerable variability in clinical practice. Uncertainties also persist regarding the optimal timing of intervention, radiation dose, and the definition of therapeutic intent, variables for which standardized criteria are still lacking.

The aim of this case report was to analyze the complex role of RT in the management of oligometastatic disease, focusing on a rare clinical scenario of metastatic bladder adenocarcinoma. This manuscript highlights the practical challenges in clinical decision-making in a setting often characterized by limited evidence and the absence of clearly established therapeutic criteria. The relevant historical context and clinical data are addressed in the Discussion section to contextualize the management of this case.

## Case presentation

We present the case of a 45-year-old male patient diagnosed with stage IV bladder cancer. His clinical history was notable for gross hematuria, which had been managed with two previous transurethral interventions without any adjuvant therapy. Subsequently, the patient developed a severe headache, prompting an evaluation that revealed synchronous metastatic dissemination to the CNS and lungs. At the time of this initial metastatic evaluation, despite the disease burden, the patient maintained a preserved performance status (Eastern Cooperative Oncology Group 1) and exhibited no significant neurological impairment beyond the headache.

To evaluate the primary bladder lesion, a transurethral biopsy was performed. Microscopic analysis of the resected specimen revealed a malignant epithelial neoplasm characterized by a proliferation of irregular papillae and glands, alongside distinct tubular structures lined by stratified columnar cells. The tumor cells exhibited oval, irregular nuclei and moderate, light eosinophilic cytoplasm. These neoplastic structures extensively infiltrated the surrounding stroma and the muscularis propria (detrusor muscle).

Although immunohistochemical profiling was not performed, a secondary or metastatic adenocarcinoma (e.g., of gastrointestinal or prostatic origin) was robustly ruled out, as the initial systemic staging workup with contrast-enhanced imaging demonstrated no evidence of an alternative primary tumor. Consequently, the correlation of these classic histomorphological features with the negative initial staging studies confirmed the diagnosis of primary muscle-invasive adenocarcinoma of the bladder.

Following the definitive diagnosis and a multidisciplinary evaluation, a sequential therapeutic plan integrating RT, immunotherapy, and surgery was established, tailored to the patient’s treatment response and clinical course.

Initial whole-brain RT (WBRT)

**Figure 1:**
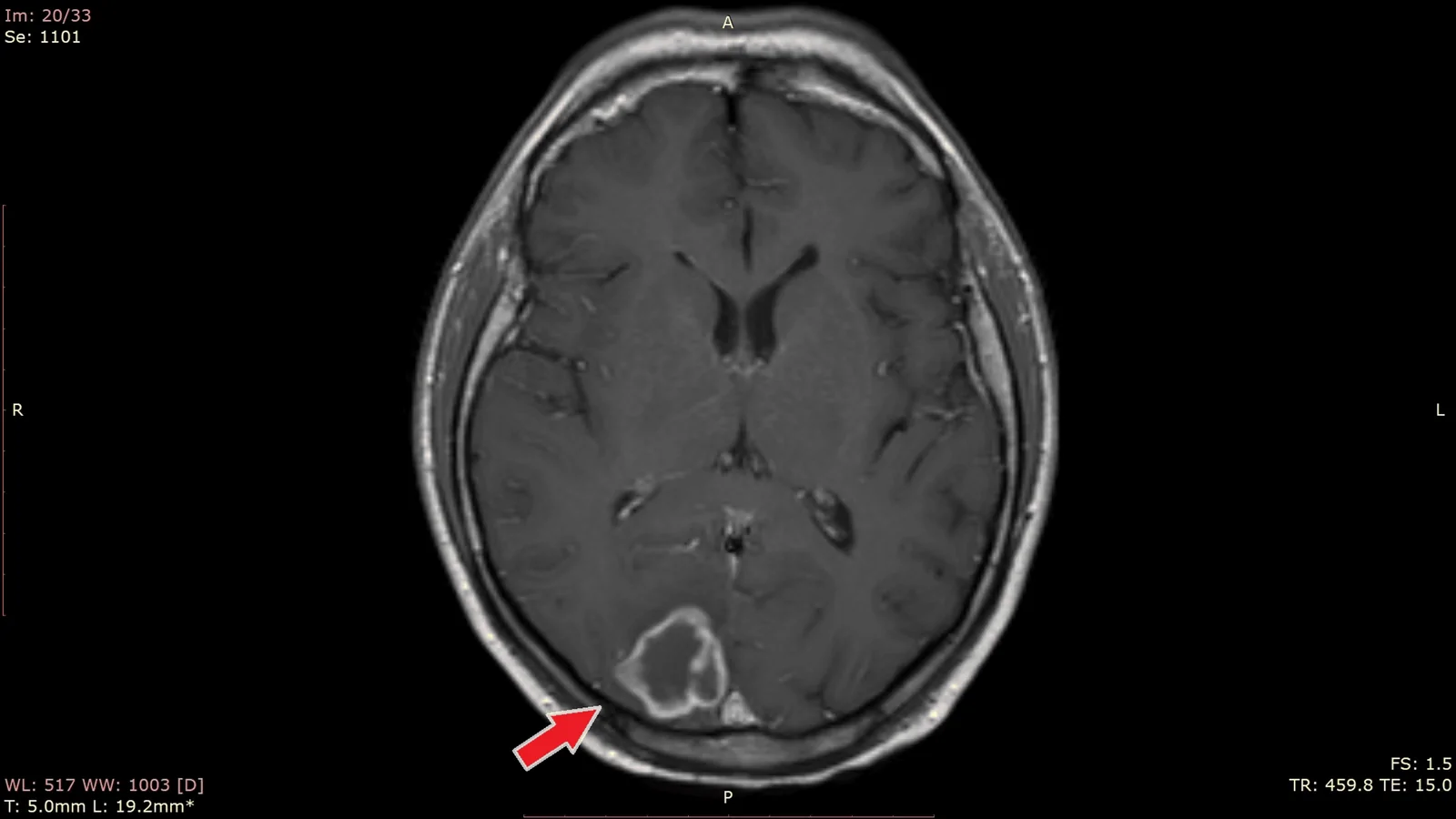
Initial 2024 MRI. T1-weighted contrast-enhanced MRI with gadolinium showing a metastatic lesion in the right occipitoparietal region (red arrow).

At the time of diagnosis, multiple brain metastases were identified, some of which were associated with perilesional edema, without prior systemic disease control. Stereotactic radiosurgery (SRS) was not considered as the initial treatment approach because of the high number of intracranial lesions, the absence of systemic control, and the clinical priority of achieving a broad therapeutic effect throughout the brain. Furthermore, this decision was pragmatically driven by the infrastructural and technological constraints at our institution at the time. Immediate access to specialized SRS planning and delivery systems was limited, making WBRT the most feasible and timely intervention to prevent acute neurological decline. Consequently, WBRT was administered at a total dose of 30 Gy in 10 fractions to ensure prompt disease control and patient safety (Figure 1).

**Figure 2:**
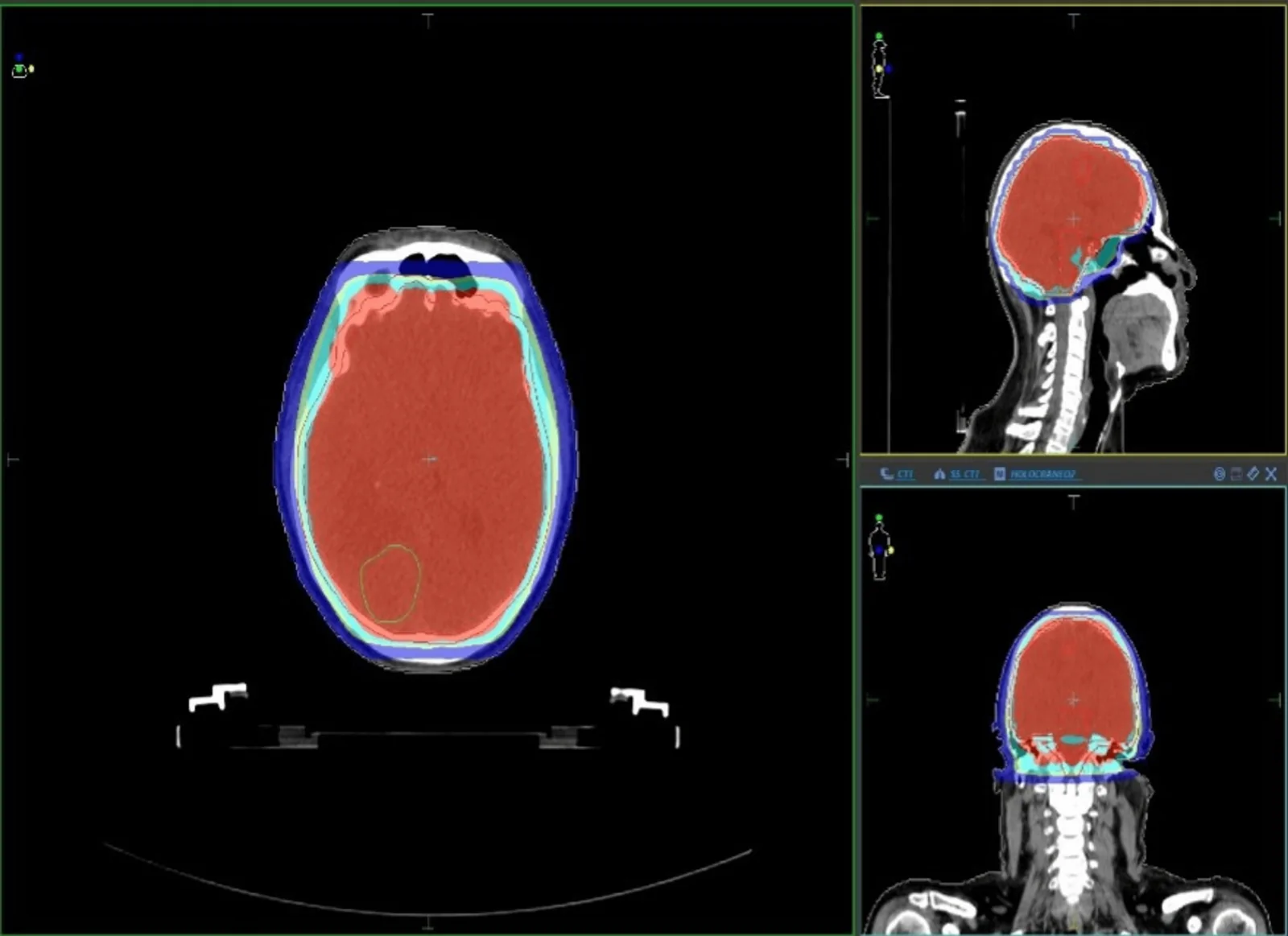
RT dose distribution. Isodose distribution in the axial, sagittal, and coronal planes of the WBRT plan generated with the MONACO system. The colors represent the 100%, 95%, 90%, and 50% isodose curves of the total prescribed dose. RT, radiotherapy; WBRT, whole-brain radiotherapy

**Figure 3:**
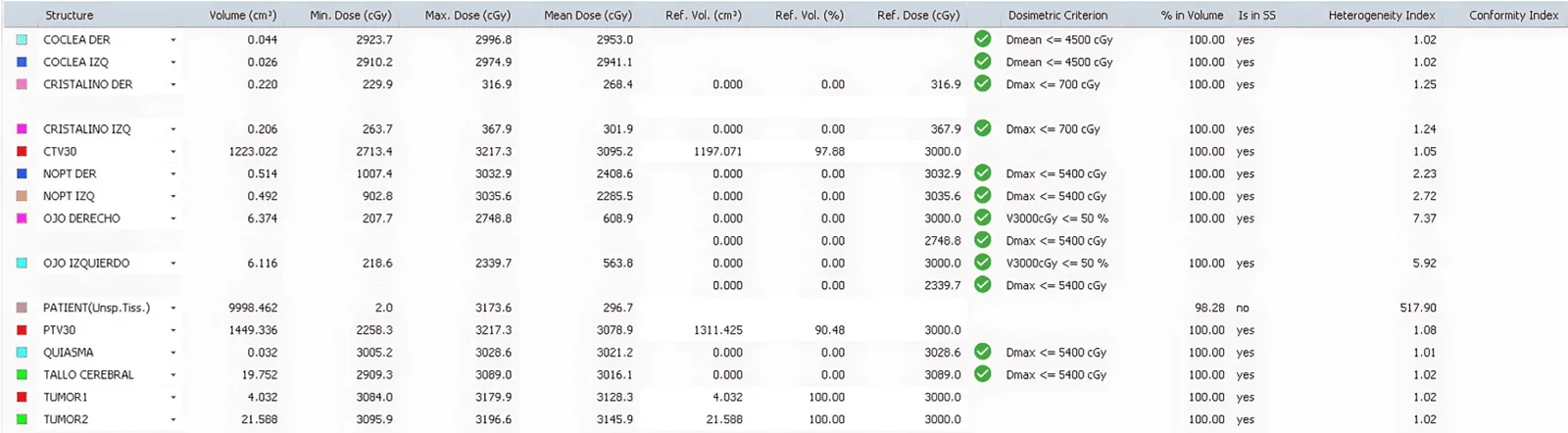
MONACO system DVH table highlighting the dosimetric constraints achieved for the OARs. DVH, dose-volume histogram; OARs, organs at risk

Treatment planning was performed using the 3D conformal RT (3D-CRT) technique with the MONACO treatment planning system, version 6.1.2.0 (Elekta, Stockholm, Sweden). The selected technique allowed safe and effective achievement of the dosimetric objectives within the institutional setting. Two opposed lateral fields at 90° and 270°, respectively, were defined using 15 MV photon beams to avoid hot spots within the patient’s scalp. In addition, multileaf collimators were closed over the orbital region in both fields to reduce the maximum dose delivered to the lenses. The contours of the planning target volume (PTV), organs at risk (OARs), and dose distribution within the treatment plan are shown in Figure 2, and the corresponding dose-volume histogram (DVH) is shown in Figure 3.

Reevaluation after initial treatment and change in clinical scenario

**Figure 4:**
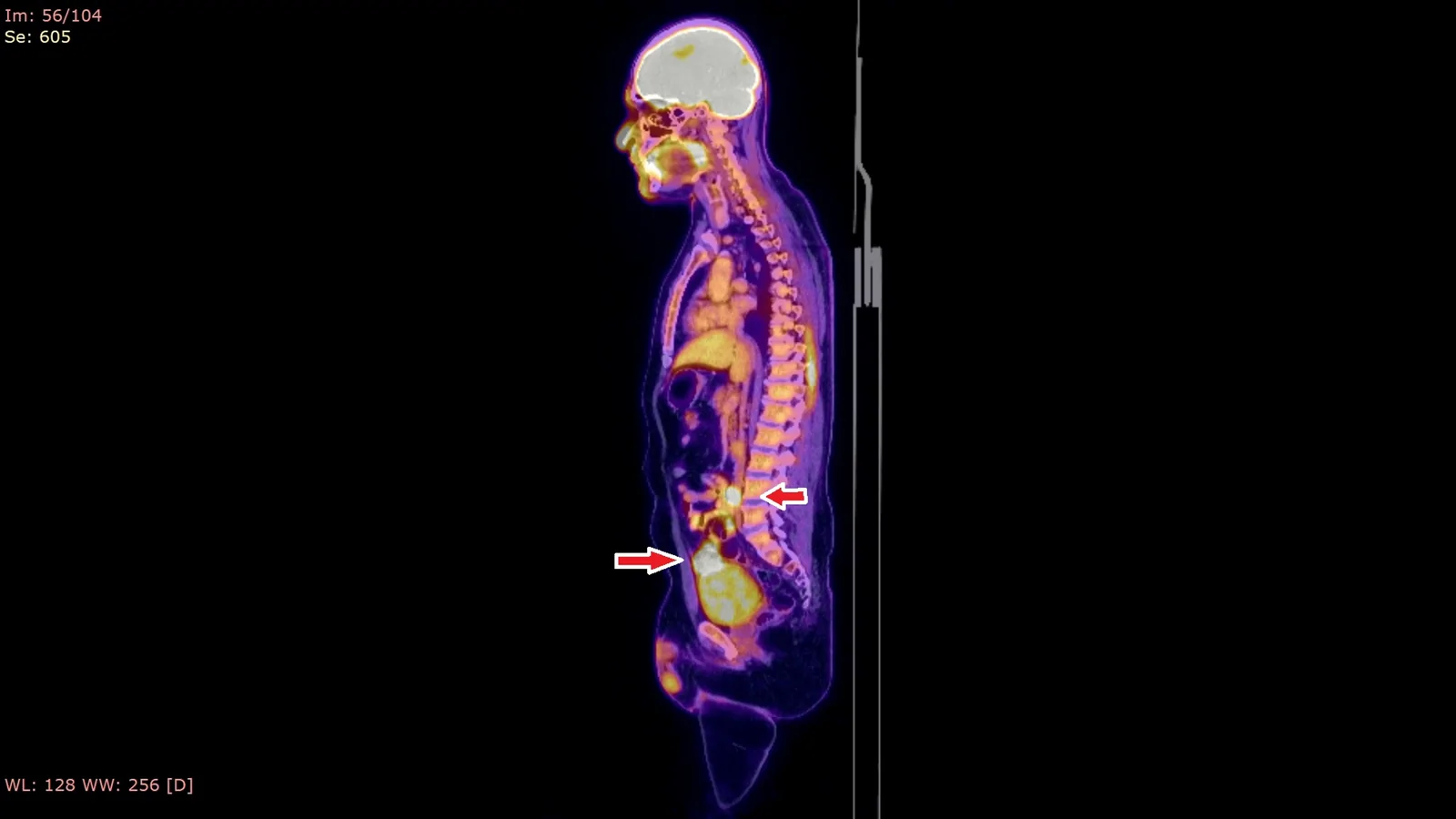
Fluorodeoxyglucose PET/CT image. The image shows fluorodeoxyglucose distribution with abnormal uptake at the bladder dome, with cranial extension of the lesion, as well as focal uptake in the common iliac lymph nodes (red arrows).

Following WBRT and initiation of pembrolizumab immunotherapy, the patient showed clinical and radiological stability of CNS disease. Reevaluation with PET/CT demonstrated disease predominantly localized to the pelvis, without significant systemic progression. In this context, the patient was no longer considered to have uncontrolled disseminated disease but rather controlled metastatic disease consistent with an oligopersistent state (Figure 4).

RT to the primary bladder tumor

Given the achieved systemic control, adequate performance status, and persistent metabolic activity at the bladder level, radical consolidative RT to the primary tumor was administered. This strategy was based on the potential benefits in local control, prevention of urinary complications, and a possible favorable impact on progression-free survival (PFS), according to contemporary evidence in selected patients with controlled metastatic disease. A total dose of 50.4 Gy in 28 fractions was delivered, with planned resimulation to allow either a boost to the primary lesion or surgical resection, if feasible. This approach corresponded to a radical-intent regimen commonly employed in neoadjuvant or organ-preserving settings, allowing an adequate balance between tumor control and toxicity while preserving the possibility of subsequent surgical intervention [3].

**Figure 5:**
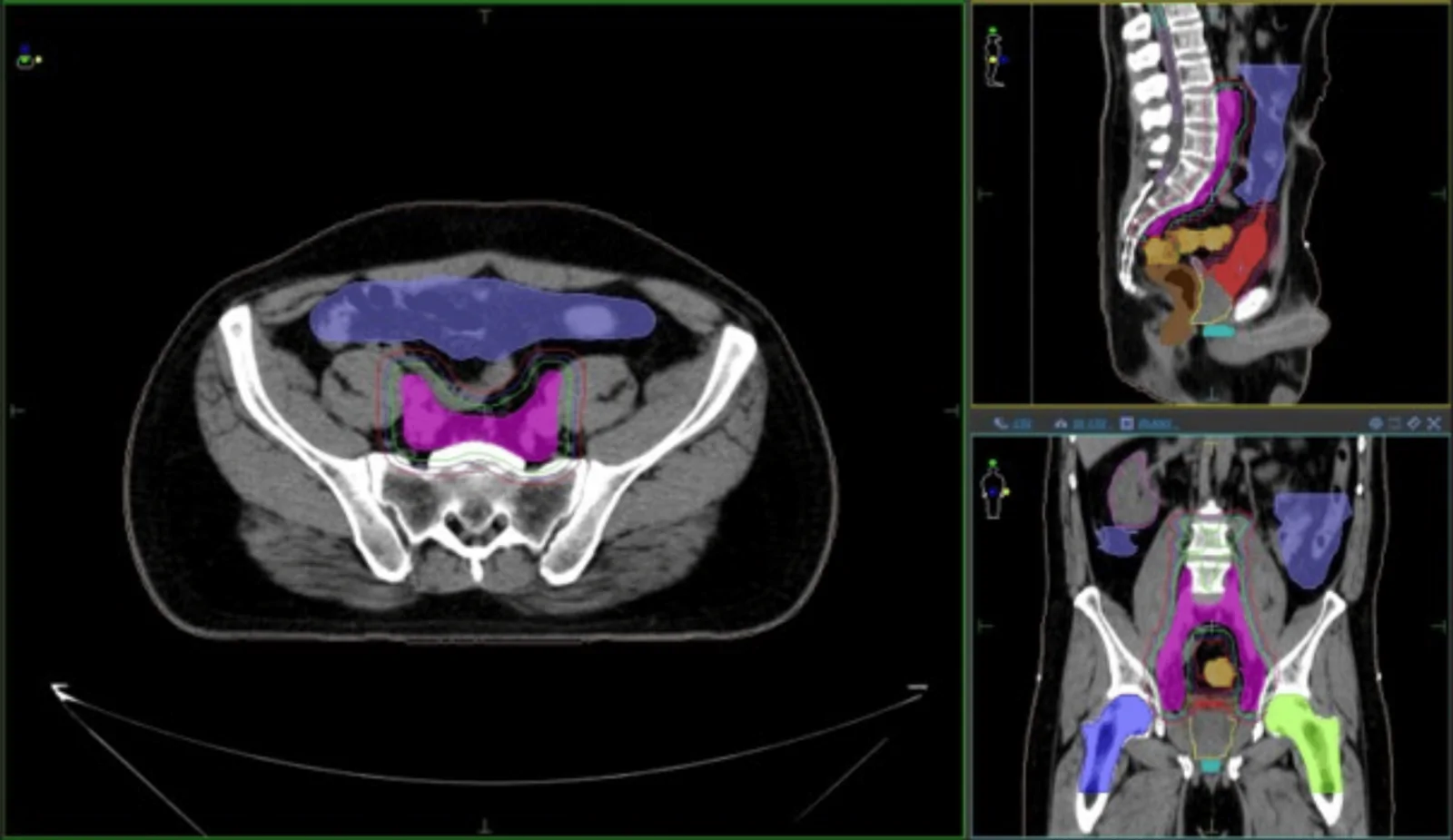
Contouring of OARs and treatment targets. The image illustrates the contours used for treatment planning. OARs, organs at risk

**Figure 6:**
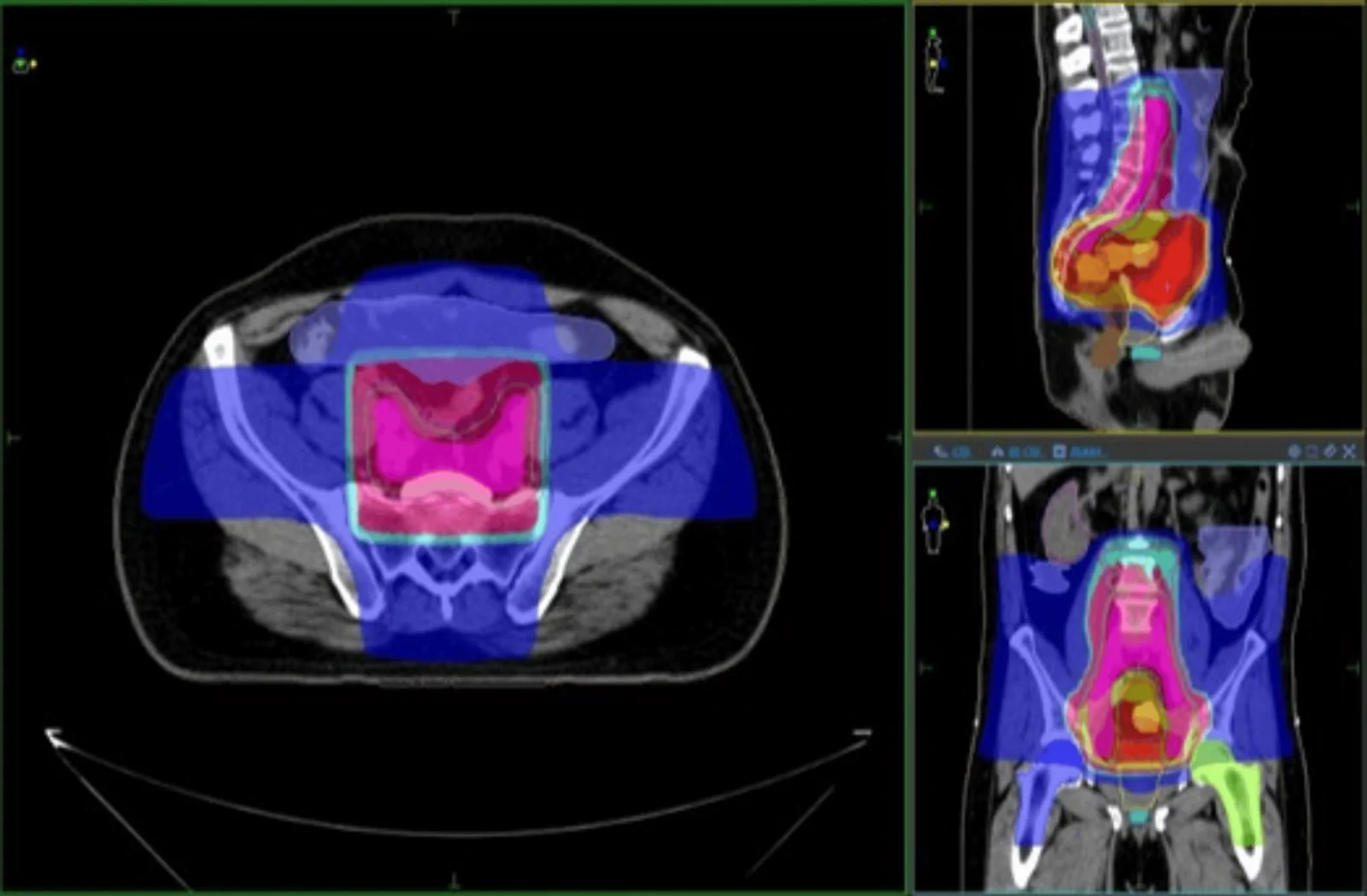
Distribution of isodose curves. Dose distribution corresponding to the prescribed dose of 50.4 Gy delivered in 28 fractions across both treatment phases. The color wash displays the isodose distributions representing 100% and 95% of the prescribed dose for both the total treatment dose (50.4 Gy) and the initial phase dose (45 Gy). Additionally, the 50% isodose distribution for the total prescribed dose is shown.

RT to the primary tumor was delivered using a 3D-CRT approach in two treatment phases. The first phase consisted of conventionally fractionated RT delivering 45 Gy in 25 fractions, followed by a 5.4 Gy boost in three fractions during the second phase. As with the WBRT treatment, planning was performed using the MONACO treatment planning system, version 6.1.2.0 (Elekta). In both phases, a four-field box technique was employed, consisting of one anterior-posterior field at 0°, one posterior-anterior field at 180°, and two lateral fields at 90° and 270°, respectively. Fifteen-MV photon beams were used for all fields, which were shaped according to the PTV defined for each phase, with 3-5 mm margins to account for setup uncertainties and internal organ motion while sparing the surrounding OARs. Field weighting was also adjusted in both phases to reduce dose exposure to the OARs (Figure 5, 6).

**Figure 7:**
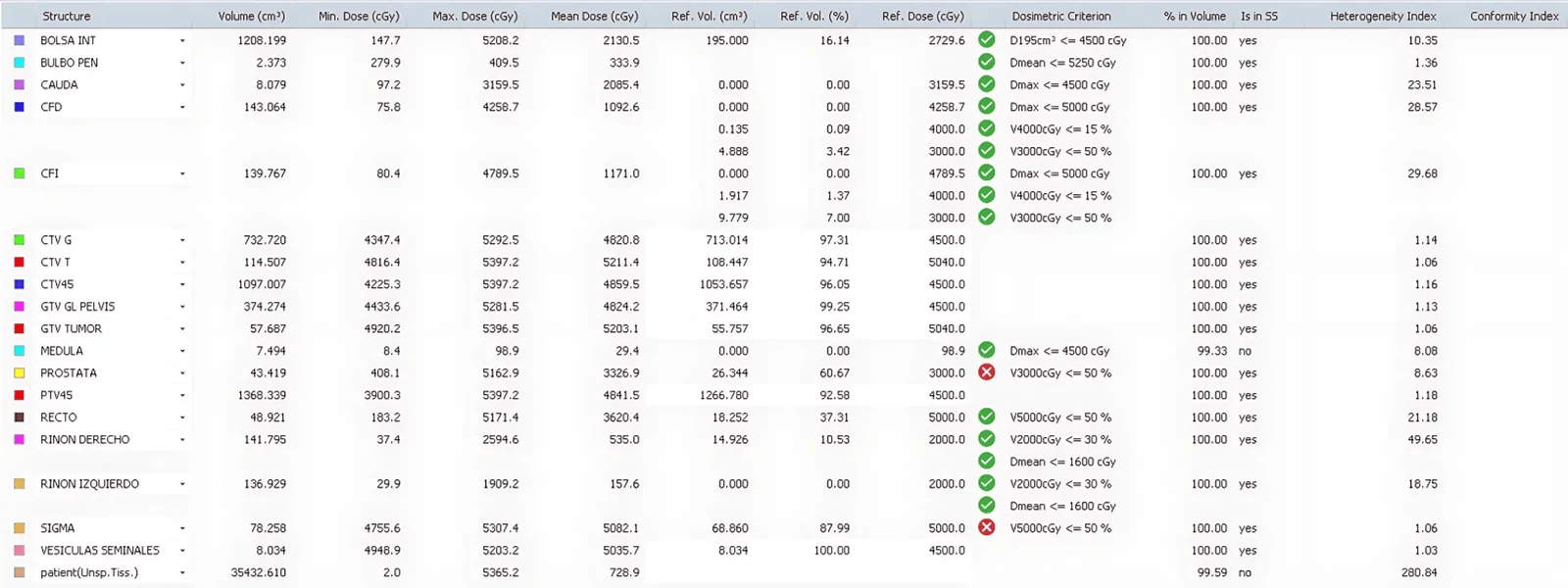
DVH table from the MONACO planning system for the pelvic plan, demonstrating the achieved constraints for the 3D-CRT technique. 3D-CRT, 3D conformal RT; DVH, dose-volume histogram

Although intensity-modulated RT and volumetric modulated arc therapy are increasingly recognized as the contemporary standard for pelvic irradiation because of their superior OAR sparing capabilities, a 3D-CRT approach was employed in this case. This decision was pragmatically driven by the technological and infrastructural resources available at our center at the time, prioritizing the timely delivery of treatment. Despite employing a standard conformal technique, meticulous dosimetric planning was performed to ensure adequate target coverage while strictly adhering to safety constraints for normal tissues. As detailed in the accompanying DVH summary table (Figure 7), the achieved doses to the rectum, bowel, bladder, and femoral heads remained well within acceptable tolerance limits, demonstrating adequate normal tissue sparing.

Subsequent surgery

After completion of RT and sustained systemic control with immunotherapy, the patient was considered a candidate for surgical resection. Given the favorable clinical and radiological response, consolidative surgery was performed. Histopathological examination of the surgical specimen revealed fibrosis and severe chronic inflammation without evidence of viable carcinoma, findings consistent with a complete pathologic response of the primary tumor.

CNS follow-up

**Figure 8:**
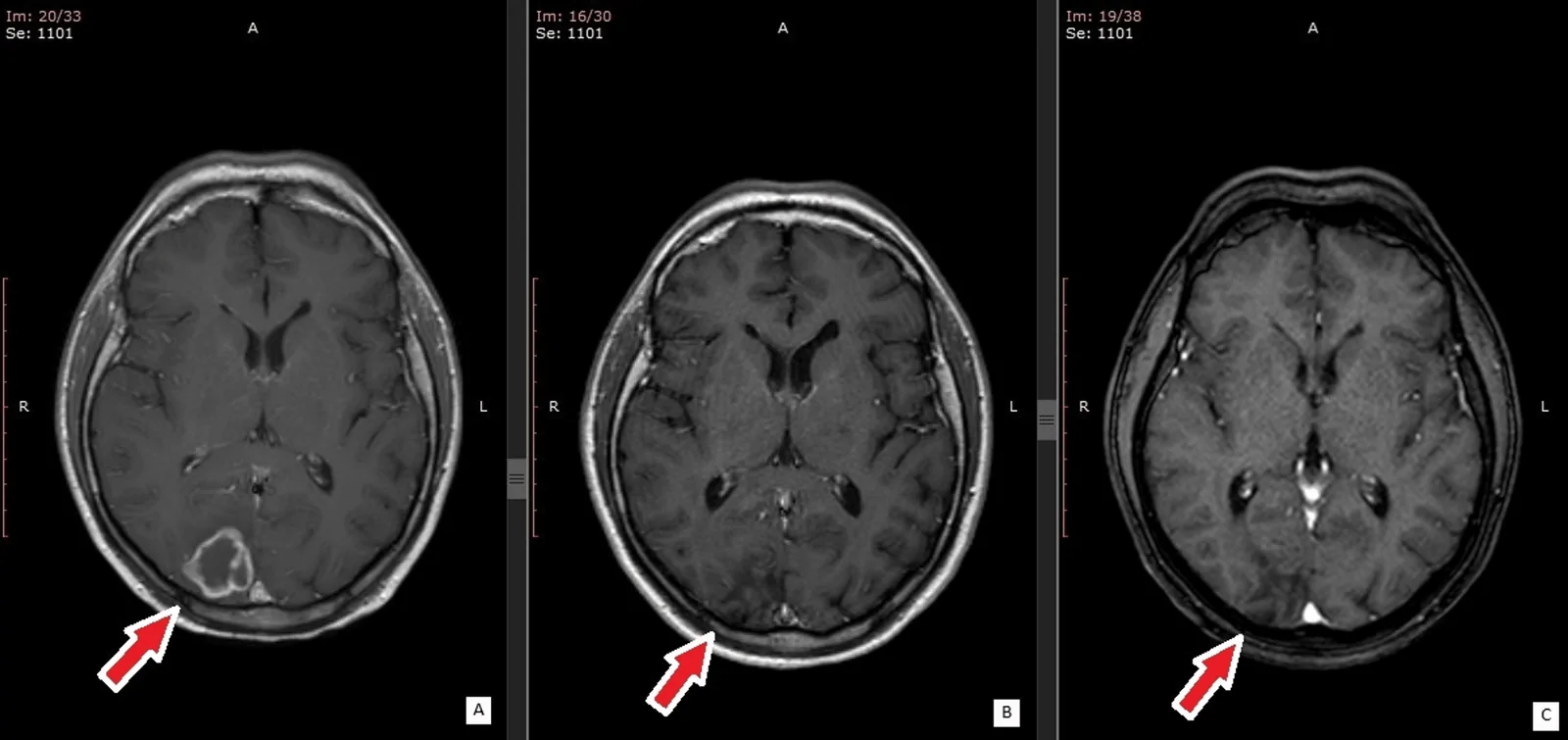
Comparative MRI. The images show post-RT follow-up in the contrast-enhanced T1 phase: from left to right, December 2024 (A), July 2025 (B), and February 2026 (C).

During follow-up, two previously irradiated residual lesions in the right frontal and occipital regions remained stable or gradually decreased in size, with an associated reduction in perilesional edema on serial MRI, without neurological symptoms or clinical deterioration (Figure 8).

Continuation of immunotherapy and clinical course

Pembrolizumab was continued as maintenance systemic therapy, and the patient completed multiple treatment cycles despite intermittent interruptions related to logistical issues rather than medical indications. Throughout follow-up, the patient remained clinically asymptomatic, with preserved functional status and no evidence of systemic progression.

Follow-up imaging, including CT of the chest, abdomen, and pelvis, showed no evidence of active systemic disease. At the latest evaluation, the patient remained under active oncologic surveillance, with local control of the primary tumor, stable brain lesions, and no evidence of active metastatic disease.

This clinical course suggests that the integration of local therapies may contribute not only to symptom control but also to consolidation of the systemic response.

## Discussion

Managing oligometastatic bladder adenocarcinoma presents a significant clinical challenge because of the scarcity of standardized guidelines. In this section, we review the historical and contemporary English-language literature to evaluate the current role of RT in this setting. Based on the clinical course of our patient, we analyze the selection criteria for local treatment, the optimal timing of intervention, therapeutic sequencing, and the integration of RT with systemic treatments.

RT in metastatic bladder cancer

Historically, metastatic urothelial cancer was managed with a predominantly palliative approach based on platinum-based chemotherapy; RT was mainly used to control hematuria, pelvic pain, and painful bone or brain metastases, commonly delivered in short-course regimens of one to five fractions [4,5]. This approach prioritized rapid symptom relief while minimizing treatment burden and toxicity in a population with a poor prognosis and fragile functional status.

Retrospective evidence has confirmed that palliative RT achieves high rates of symptom control, particularly for hematuria and pain, with a generally acceptable toxicity profile. Therefore, it remains a cornerstone of management in patients with extensive disease, poor functional status, or those who are not candidates for systemic therapy [4,5].

However, this paradigm was based on the premise that RT only influenced local symptom control without modifying the natural history of the disease. This perspective has progressively been reevaluated owing to advances in imaging and staging techniques, improved systemic disease control, and a deeper understanding of the biological heterogeneity of metastatic disease.

Evolution of the role of RT in the era of modern systemic therapies

Improvements in imaging techniques and the incorporation of more effective systemic therapies, including cisplatin-based regimens, avelumab maintenance, immune checkpoint inhibitors (ICIs), fibroblast growth factor receptor inhibitors, and antibody-drug conjugates, have changed the management of metastatic bladder cancer. These advances have allowed a growing number of patients to achieve prolonged systemic control while maintaining a limited metastatic burden, supporting the recognition of oligometastatic disease (≤3-5 lesions) as a clinical state in which local interventions with radical intent may be integrated into the therapeutic strategy. The development of stereotactic body RT (SBRT) has further expanded the use of locoregional treatments with consolidative or radical intent in carefully selected patients [4,6,7].

Retrospective evidence and recent meta-analyses suggest that RT directed at the primary tumor and/or residual metastatic lesions may be associated with improved oncologic outcomes in selected patients with oligometastatic urothelial cancer. Retrospective series and systematic reviews have demonstrated high local control rates and low toxicity with metastasis-directed RT, particularly with SBRT [7,8]. Likewise, Aboudaram et al. reported prolonged PFS and overall survival (OS) in patients with ≤5 residual lesions without progression after first-line systemic therapy who were treated with consolidative RT to the bladder tumor and metastatic sites, reporting a hazard ratio of 0.49 for PFS and 0.47 for OS, with a favorable safety profile and only 3.9% grade 3 toxicity [9]. Nevertheless, it must be acknowledged that their cohort consisted almost entirely of patients with de novo or genuine oligometastatic disease. In contrast, our patient represents an induced oligopersistent state, having initially presented with widely disseminated synchronous metastases (including pulmonary and brain lesions) that partially resolved following systemic therapy. Biologically, induced oligopersistent disease is a distinct entity that historically carries a poorer prognosis than genuine oligometastatic disease. Because direct evidence guiding the management of this specific, higher-risk subgroup remains scarce, the durable clinical control achieved in this patient highlights the potential value of extending consolidative local therapies to carefully selected patients with oligopersistent disease.

Similarly, population-based studies suggest a potential benefit of RT directed to the primary bladder tumor even in the presence of metastatic disease. An association has been observed between bladder-directed RT combined with systemic chemotherapy and improved OS compared with chemotherapy alone in patients presenting with metastatic disease at diagnosis, although these findings are limited by the retrospective design and potential selection bias [10]. Meanwhile, prospective trials such as GETUG-AFU V07/BLAD-RAD01 are evaluating consolidation strategies incorporating bladder RT and ablative RT for limited residual disease in addition to avelumab maintenance following initial systemic therapy, with the aim of defining their impact on oncologic outcomes in patients with a low metastatic burden [11].

The combination of RT and immunotherapy is an area of growing interest in metastatic bladder cancer because of potential synergistic biological mechanisms. Radiation induces immunogenic cell death, promotes the release of tumor antigens, and activates the cGAS-STING pathway, which is essential for type I interferon production and dendritic cell maturation. At the same time, radiation increases the expression of major histocompatibility complex class I (MHC-I) molecules and adhesion ligands such as ICAM-1, thereby optimizing the priming and infiltration of cytotoxic T lymphocytes. By integrating ICIs, such as anti-PD-1/PD-L1 agents, the exhausted state of T cells may be reversed, enhancing not only local control but also the regression of distant lesions, representing the highest level of this synergistic interaction. The combination of these modalities may transform previously resistant or “cold” tumors by increasing MHC-I expression and activating the cGAS-STING pathway, converting them into inflamed or “hot” tumors that may respond better to immunotherapy [12,13].

Although its clinical relevance in bladder adenocarcinoma remains uncertain and has not yet been consistently demonstrated in prospective studies, the available evidence continues to be derived mainly from retrospective and early-stage studies. Nevertheless, contemporary reviews support the clinical feasibility and therapeutic potential of combining RT and immunotherapy in selected patients with locally advanced or metastatic bladder cancer [12,14].

Rationale and scenarios beyond pure palliation

RT in metastatic bladder cancer is no longer regarded exclusively as a palliative modality for symptom control but is now increasingly being integrated into consolidation strategies and biologic control approaches for oligometastatic disease. This conceptual shift arises from the observation that a subgroup of patients with a low metastatic burden exhibits a less aggressive clinical course and may potentially benefit from local ablative therapies directed at all visible lesions. In these settings, SBRT has demonstrated high rates of local control, favorable toxicity profiles, and the potential to delay systemic progression and the need for additional lines of systemic therapy [7].

The underlying biological hypothesis proposes that certain metastases act as active reservoirs of continuous tumor dissemination; therefore, their eradication could limit subsequent clonal spread and partially modify the natural history of the disease. Multicenter retrospective studies have shown that the clinical benefit appears to be concentrated in carefully selected patients, particularly those with solitary metastases, metachronous metastases, a low tumor burden, and good performance status, suggesting that not all oligometastatic states share the same tumor biology [8]. In fact, Svedman et al. identified a subgroup of patients with a solitary metastatic lesion who achieved prolonged survival without requiring subsequent systemic therapy after SBRT, suggesting the existence of a true oligometastatic phenotype that could benefit from local ablative strategies [15].

Furthermore, in oligoprogressive settings, SBRT allows eradication of acquired clonal resistance foci while maintaining otherwise effective systemic therapy against the remaining disease, thereby delaying the need to transition to the next line of therapy [8]. Although the available evidence remains limited by the predominance of retrospective studies and the inherent selection bias of these cohorts, current findings support the incorporation of ablative RT into multimodal treatment strategies for selected patients with metastatic urothelial carcinoma [8,15].

Additionally, the timing of RT directly influences its clinical utility. Available data suggest more favorable outcomes when RT is delivered after a documented response or stable disease following systemic therapy and in the presence of limited residual disease, rather than as an upfront intervention in uncontrolled metastatic disease [4,5,9].

The multidisciplinary approach to cancer care has been, and should remain, a cornerstone for defining therapeutic strategies and clinical objectives in individual cases. Current knowledge has evolved significantly, and a broader therapeutic spectrum is now considered in the management of these patients. The integration of molecular biology, targeted therapies, advanced RT techniques, and surgery has expanded the conceptual framework of oligometastatic disease and supports the inclusion of carefully selected patients within this paradigm. Although this intermediate state between localized and disseminated disease has been defined [16], it remains essential not to miss opportunities for therapeutic intervention that may alter the treatment paradigm and improve patient outcomes through enhanced local control and OS, particularly given the consistently favorable trends reported in the available evidence [17,18].

Toward a management algorithm for oligometastatic and oligopersistent disease

**Figure 9:**
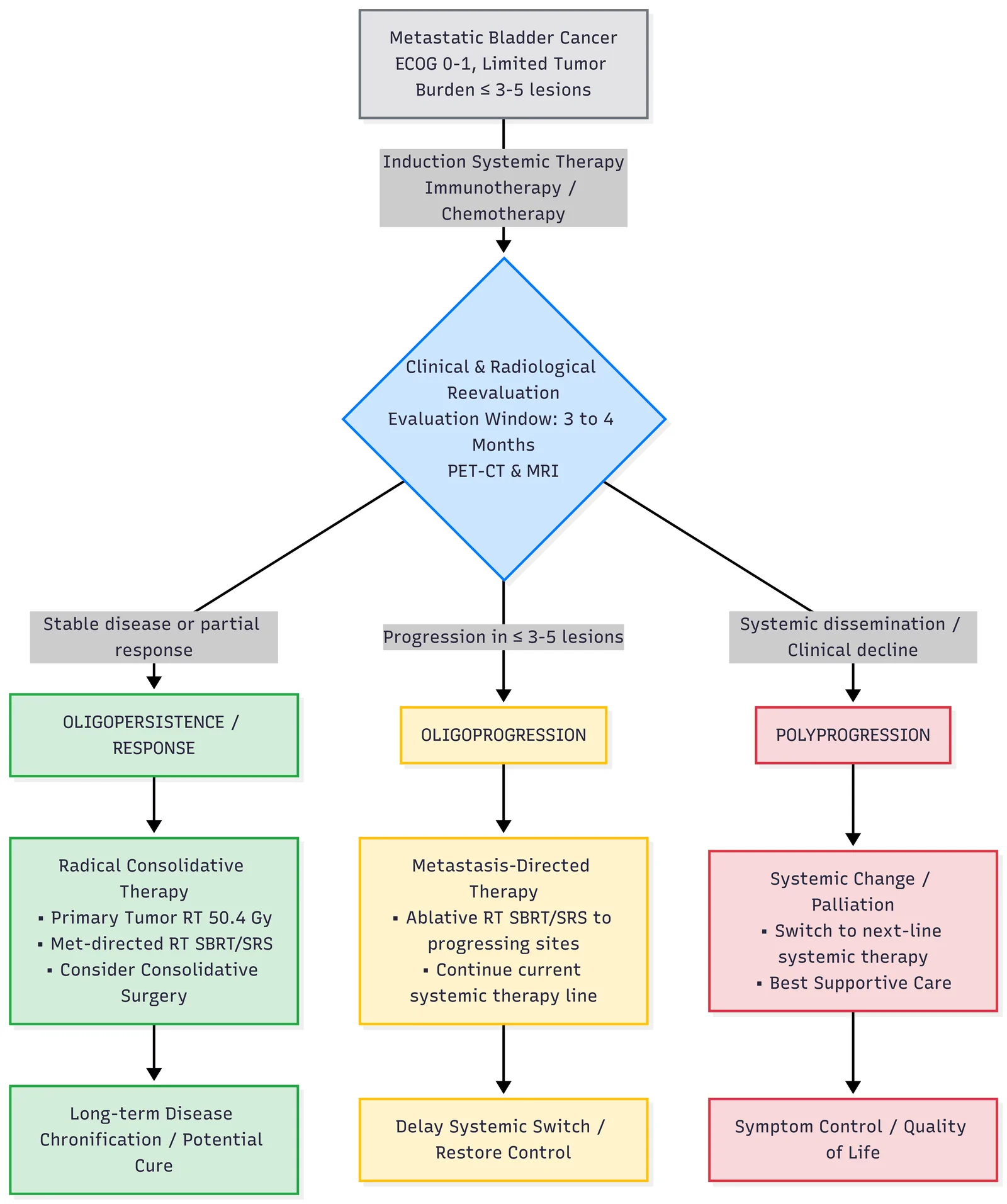
Therapeutic algorithm for managing oligometastatic and oligopersistent disease. ECOG, Eastern Cooperative Oncology Group; RT, radiotherapy; SBRT, stereotactic body radiotherapy; SRS, stereotactic radiosurgery This figure was created using Mermaid Live Editor, developed by the Mermaid Open Source Project (Stockholm, Sweden).

Clinical decision-making in patients with advanced bladder cancer is highly complex, requiring a delicate balance between systemic control and the optimal timing of local intervention. Based on the available evidence and the clinical trajectory observed in this case, we propose a redefined decision-making framework (Figure 9) that shifts the focus from a purely reactive model to a proactive strategy of early interception, aiming for the long-term precision chronification of the disease.

The cornerstone of this approach is the temporal sequencing of therapies. Contemporary consensus, including frameworks from the European Society for Radiotherapy and Oncology and the European Organisation for Research and Treatment of Cancer [16], suggests that aggressive local therapies should not be universally applied upfront in the metastatic setting. Instead, an initial induction phase with systemic therapy, typically lasting three to four months (or three to six cycles), is highly recommended [4,9]. This induction period serves as a critical in vivo biological test. It effectively filters out patients with aggressive, rapidly progressing micrometastatic disease who would not benefit from, and might be harmed by, radical local interventions [5,18].

For patients who achieve disease stability or a partial response following this systemic induction, the clinical scenario transitions into an oligopersistent state [16]. It is within this specific window of opportunity that consolidative local therapy becomes paramount. As demonstrated in our patient, who received radical-intent RT to the primary tumor (50.4 Gy) after achieving systemic stability, targeting the residual active foci can eradicate treatment-resistant clones before they seed further dissemination.

Consequently, the therapeutic algorithm must clearly distinguish between different clinical states after the initial systemic evaluation. In patients with oligopersistent disease or a favorable response, radical consolidative RT to the primary tumor and residual metastases offers a pathway to complete pathologic response and durable disease control [9,10]. Conversely, in oligoprogressive disease, metastasis-directed therapy, such as SBRT or SRS, is used to ablate isolated resistant clones while maintaining the current effective systemic therapy line [8,15].

## Conclusions

This case illustrates the feasibility of a sequential radical approach in metastatic bladder adenocarcinoma, which may contribute to durable disease control in appropriately selected patients following an initial systemic response. Furthermore, the integration of modern RT techniques with immunotherapy adds to the growing evidence supporting a shift from a purely palliative strategy toward a consolidative approach, highlighting a promising avenue for optimizing survival outcomes in the oligometastatic setting that warrants further investigation.
